# Complete Genome Sequence of *Spirosoma* sp. Strain KCTC 42546, Isolated from a Reservoir in South Korea

**DOI:** 10.1128/MRA.00694-20

**Published:** 2020-09-17

**Authors:** Pokchut Kusolkumbot, Song-Gun Kim, Chatrudee Suwannachart

**Affiliations:** a Biodiversity Research Centre, Thailand Institute of Technological Research, Pathum Thani, Thailand; b Biological Resource Center, Korean Collection for Type Cultures, Korea Research Institute of Bioscience and Biotechnology, Ipsingil, Jeongeup, Jeonbuk, Republic of Korea; Portland State University

## Abstract

This study reports on the complete genome sequence of *Spirosoma* sp. strain KCTC 42546, isolated from fresh water in a reservoir in South Korea. The genome contained genes for various glycosyl hydrolases, which are associated with degrading sugars and DNA-repairing enzymes.

## ANNOUNCEMENT

The genus *Spirosoma* was recognized with Spirosoma linguale as the type species and included in the approved list of bacterial names (1, 2). *Spirosoma* species have been discovered to exist in dust (3), fresh water (4), soil (5, 6), and extreme environments such as high Arctic glaciers (7). The genus contains strains with high activity of glycosyl hydrolases, including α-l-fucosidase (GH29) and xylan-β-1,4-xylosidase (GH39) cellulase or hemicellulases, which degrade polysaccharides of plant origin (8). The genus *Spirosoma* also comprises strains with high radiation resistance (9). Many unique enzymes for DNA repair have been found in the genome of Spirosoma pulveris JSH 5-14^T^ (9). *Spirosoma* sp. strain KCTC 42546 was isolated from fresh water collected from the Daecheong Reservoir, South Korea (36°22′25″N, 127°28′35″E). This reservoir was artificially made to generate electricity and to provide water for the people and industries in the Daejeon and Chungcheong areas. Serial dilutions of the sample in 0.85% NaCl were plated onto Reasoner’s 2A (R2A) agar and incubated at 25°C (9). Single colonies were transferred onto an R2A agar plate and incubated for 3 days at 25°C (9). *Spirosoma* sp. strain KCTC 42546 was cultivated for 3 days in 100 ml R2A agar at 25°C under agitation (180 rpm). Genomic DNA (gDNA) was extracted using a MagListo 5M genomic DNA extraction kit (Bioneer, South Korea) following the manufacturer’s protocol. The DNA quality and quantity were determined using a NanoDrop spectrophotometer (Thermo Scientific, USA). Default parameters were used for all software unless otherwise specified. SMRTbell DNA libraries were prepared using the PacBio P6 DNA polymerase binding kit and DNA template preparation kit 3.0 (Pacific Biosciences, Menlo Park, CA, USA) according to the manufacturer’s protocol. The Pacific Biosciences RS II (PacBio) system was used with P6-C4 chemistry on one single-molecule real-time (SMRT) v2.3 cell per genome. In total, 152,504 PacBio subreads with 1,678,365,911 bp were generated, and their mean length and *N*_50_ value were 11,005 and 15,939 bp, respectively. *De novo* assembly was performed with the Hierarchical Genome Assembly Process v3 (HGAP3), and error correction was conducted using Quiver (3). The total genome was 8,662,329 bp with a G+C content of 47.43%. The genome was larger than those of *S. linguale* and *S. pulveris* (5, 7). The assembled contigs were annotated using Non-supervised Orthologous Groups (eggNOG). The eggNOG annotation was performed using the BLAST software with the eggNOG v4 database (10). The circular genome consisted of 8,246 genes, including 6,896 coding DNA sequences (CDS), 45 tRNA genes, and 9 rRNA genes.

### Data availability.

This whole-genome sequence of a novel *Spirosoma* sp. isolate has been deposited in GenBank under accession no. CP041360. The BioProject and BioSample accession no. are PRJNA522343 and SAMN10928934, respectively.
